# Case Report: A Rare Case Example Of Charles Bonnet Syndrome

**DOI:** 10.1192/j.eurpsy.2025.2217

**Published:** 2025-08-26

**Authors:** S. Göktaş, F. Monshizadeh Tehrani, E. Ilgın, A. Sakallı Kani

**Affiliations:** 1 Department of Psychology, Yeditepe University, Istanbul, Türkiye; 2 Child and Adolescents Mental Health Center, Mental Health Services in the Capital Region of Denmark, Copenhagen, Denmark; 3 Department of Psychiatry, Marmara University, Istanbul, Türkiye

## Abstract

**Introduction:**

Charles Bonnet syndrome, first described by Charles Bonnet in 1760, is characterized by visual hallucinations in cognitively normal patients who are typically elderly and often visually impaired.

**Objectives:**

This case has been evaluated as a typical Charles Bonnet syndrome characterized by multisensory hallucinations (visual, auditory, olfactory hallucinations) observed in a patient with NF-2 diagnosis. In this respect, this case analysis might make an important contribution to the literature, as it might expand the understanding of CBS in the context of complex neurological disorders. Consideration must be put into the possibility of associating the patients’ multisensory hallucinations with organic pathology due to the presence of occipital and temporal meningiomas.

**Methods:**

In this section, data obtained from our own case will be included. Additionally, a literature review was conducted using PubMed, restricting the search to studies published between 2014 and 2024. The search term “Charles Bonnet Syndrome” was used to identify relevant articles. Furthermore, a detailed anamnesis of the disease process was obtained from the patient’s mother.

**Results:**

A 31-year-old female first presented to our psychiatry outpatient clinic in July 2024, with her relative reporting a diagnosis of neurofibromatosis type 2. It was documented that the patient underwent surgery in November for diffuse meningiomas (both supra- and infratentorial, involving the temporal and occipital regions). Also, the patient experienced total hearing loss due to vestibular schwannomas in the right region and suffered from visual loss during the pre-operating period. She reported a distressing increase in the acute perception of the smell and taste of food and beverages. Her first psychiatric examination was conducted postoperatively. Olanzapine 2.5mg/day was commenced as a treatment for relative auditory and olfactory hallucinations, and the dose of the drug was gradually increased to 10mg/day. The final examination was conducted on August 26, 2024. According to reports from the patient’s relatives, there was a noted decrease in the frequency and intensity of the hallucinations. Throughout the medical examinations, communication with the patient was partially facilitated using tactile cues.

**Conclusions:**

A typical Charles Bonnet syndrome is a disorder characterized by visual loss accompanied by visual hallucinations. In this case, olfactory, auditory and alpha-factor hallucinations that developed after visual and hearing loss were detected and resulted in the involvement of more than one sensory organ, shedding light on the current literature. In this case, the fact that the person already had neurofibromatosis type 2 disease further complicates the etiology of these symptoms and requires detailed follow-up and treatment.

**Disclosure of Interest:**

None Declared

